# Lymph node ratio as a prognostic factor in patients with pathological N2 non-small cell lung cancer

**DOI:** 10.1186/s12957-016-1048-5

**Published:** 2016-11-25

**Authors:** Masaya Tamura, Isao Matsumoto, Daisuke Saito, Shuhei Yoshida, Munehisa Takata, Hirofumi Takemura

**Affiliations:** Department of Thoracic, Cardiovascular and General Surgery, Kanazawa University School of Medicine, Takara-machi 13-1, Kanazawa, 920-8640 Japan

**Keywords:** Lung cancer surgery, Mediastinal lymph nodes, Metastases (lymph node)

## Abstract

**Background:**

The aim of this study was to investigate whether the lymph node ratio (LNR) was associated with the prognosis of patients, who underwent surgery for pathological N2 non-small cell lung cancer (NSCLC).

**Methods:**

A total of 182 patients were diagnosed with pathological N2 disease and underwent complete resection surgeries with systematic lymphadenectomies. We counted the number of positives and removed lymph nodes to calculate a ratio between them (LNR). We also investigated the association between skip mediastinal lymph node metastasis and survival.

**Results:**

Univariate analysis of survival in patients with N2 NSCLC showed that the T factor, clinical N factor, and LNR were significant prognostic factors. Multivariate analyses showed that the clinical N stage and LNR were significant independent prognostic factors for patients with pathological N2 NSCLC. Patients with a clinical lymph node status of 0 (cN0) and LNR ≤0.22 showed a significantly higher survival rate than patients with a cN1-2 and LNR ≥0.22 and 5-year survival rates were 47.1 and 10.3%, respectively (*p* < 0.0001).

**Conclusions:**

LNR is an important prognostic factor for poor outcome following surgery in patients with N2 disease. The combination of the LNR and cN status provides a valuable prognostic tool.

## Background

Non-small cell lung cancer (NSCLC) featuring clinical mediastinal disease is not often amenable to complete resection. The survival range among patients with stage III NSCLC is associated with various prognostic factors, suggesting that patients at the N2 stage are a heterogeneous group [1, 2]. The heterogeneity of NSCLC involves multiple factors, including preoperative detection, neoadjuvant therapy susceptibility, clinically unsuspected N2 disease (the presence of ipsilateral mediastinal nodal metastases), and the level or site and number, or both, of involved mediastinal lymph nodes [1, 3]. Few reports have evaluated the lymph node ratio (LNR) as a prognostic factor for N1 and N2 NSCLC [4, 5]. Therefore we investigated the correlation between LNR and prognosis in patients with pathological N2 NSCLC.

## Methods

### Patients

The hospital records of 1839 consecutive patients who underwent a complete NSCLC resection between 1990 and 2010 were reviewed and 227 patients with pathological N2 disease were identified. Patients were excluded from the study if they underwent segmentectomy or wedge resection of the lung and the number of resected lymph nodes was ≤6, and patients who died within 30 days of surgery or without enough clinicopathological and prognostic information were also excluded. A total of 182 patients (127 men and 55 women), ranging in age from 36 to 89 years (median age, 66 years), were included in this study. All of these 182 patients underwent initial operation. Patients were diagnosed as having pathological N2 who underwent surgically complete resection with a systematic lymphadenectomy. Lymph node metastasis was preoperatively diagnosed using computed tomography (CT) scan. Since 2004, we introduced fluoro-deoxyglucose-positron emission tomography (FDG-PET), which was used as a reference and performed on 48 patients. Mediastinal lymph nodes with a short axis of >1 cm (especially >1.5 cm for Station. 7) on CT and/or positive uptake on FDG-PET were regarded as metastatic lymph nodes. Lymph node biopsy through mediastinoscopy was not performed routinely and was performed selectively in patients with clinical N2. Twenty-eight of 182 patients were performed mediastinoscopy and 17 patients were diagnosed as having clinical N2. For patients with clinical single-level N2 disease, we elected to perform initial operation. Magnetic resonance imaging (MRI) was routinely employed for brain metastasis assessment. Patients who died within 1 month after surgery or received chemoradiotherapy before surgery were excluded from the study. Follow-up information was obtained from all patients through outpatient visits or telephone interviews either with the patients, their relatives, or primary physicians. The outcomes included the type of recurrence and survival time. Patient demographics and tumor characteristics are detailed in Table 1.

**Table 1:** Characteristics of patients with pathological N2 disease

Variables		Number of patients	%
Age (years), range		36–89	
	Mean	64.6	
Gender			
	Male	127	69.8
	Female	55	30.2
Histology			
	Adenocarcinoma	120	65.9
	Squamous cell carcinoma	50	27.5
	Others	12	6.6
Clinical T and N status			
	T1 N0	49	26.9
	T2 N0	51	28.0
	T3 N0	3	1.7
	T4 N0	2	1.1
	T1 N1	5	2.8
	T2 N1	11	6.0
	T3 N1	3	1.6
	T1 N2	21	11.5
	T2 N2	25	13.8
	T3 N2	12	6.6
Clinical node (cN) factor			
	cN0	105	57.7
	cN1	19	10.4
	cN2	58	31.9
Pathological tumor (pT) factor			
	pT1	59	32.4
	pT2	92	50.6
	pT3	31	17.0
Side			
	Right	101	55.5
	Left	81	44.5
Number of metastatic stations			
	Single	56	30.8
	Multiple	126	69.2
Skip N2			
	Skip	69	37.9
	Non-skip	113	62.1
Operative procedure			
	Pneumonectomy	15	8.3
	Bilobectomy	19	10.4
	Lobectomy	148	81.3
Adjuvant chemotherapy			
	Yes	140	76.9
	No	42	23.1

### Lymph node ratio and lymph node dissection

Pathologists counted the number of lymph nodes by observing the membrane integrity, which meant that several parts of the lymph node tissue were countered as one lymph node. We counted the number of positive and removed lymph nodes to calculate the LNR and investigated the association between skip mediastinal lymph node metastasis, which is defined as the mediastinal lymph node metastasis without hilar lymph node metastasis and survival. Five-year survival of high and low LNR groups was calculated. Maximum difference of 5-year survival could be available when we set LNR of 0.22 as a cut-off.

### Pathological examination

After localization and size measurement, the specimens were serially sectioned (3–4 mm) using a cryostat embedded and stained with standard hematoxylin and eosin. The tumor node metastasis (TNM) stage was assigned according to the American Joint Committee on Cancer staging system, seventh edition. All patients demonstrated macroscopically negative surgical margins.

### Follow-up

Follow-up examinations included chest X-rays and blood tests at 3-month intervals and an additional thoracic CT scans at 6-month intervals. The median follow-up duration was 42 months (range, 12–127 months).

### Statistical analysis

All data regarding continuous variables are expressed as mean ± SD. Significant differences were assessed using the *t* test for continuous variables and the *χ* ^2^ test for categorical variables. Outcome measures included type of recurrence and survival time. Analyses were performed using the SAS software package (SAS Institute, Inc, Cary, NC). A *p* value of <0.05 was considered statistically significant.

## Results

**Fig. 1:**
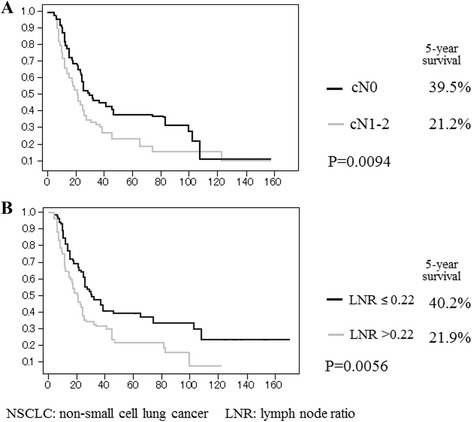
Probability of survival of patients with N2 non-small cell lung cancer according to clinical lymph node status (**a**) and lymph node ratio (**b**)

**Fig. 2:**
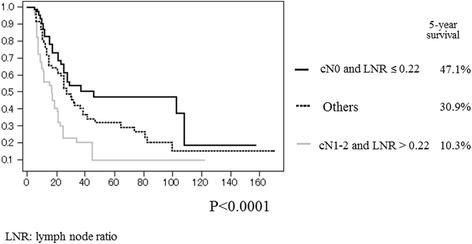
Overall survival rate for patients with N2 non-small cell lung cancer depends on clinical lymph node status (cN) and lymph node ratio (LNR). cN0 and LNR ≤0.22 (*black line*). cN0 and LNR >0.22, or cN1-2 and LNR ≤0.22 (*black dashed line*). cN1-2 and LNR > 0.22 (*gray line*)

Patient characteristics are summarized in Table 1. The patients included 127 males and 55 females. Pathological types included 120 adenocarcinomas, 50 squamous cell carcinomas, and 12 other types of NSCLC. Clinical N lymph node (cN) stages were diagnosed as N0 in 105 patients, N1 in 19, and N2 in 58. There were 59 patients diagnosed as T1, 92 as T2, and 31 as T3. Skip mediastinal lymph node metastasis (N1 negative) was demonstrated in 69 patients (37.9%), and mediastinal lymph nodes metastasis with N1 disease (N1 positive) was found in 113. A pneumonectomy was performed in 15 (8.3%) patients, bilobectomy in 19 (10.4%), and lobectomy in 148 (81.3%). The median number of removed nodes was 21, and the median number of positive nodes was 3. The median LNR was 0.24. A univariate analysis of survival in patients with N2 NSCLC showed that the T factor (T1 or 2 vs. T3, *p* < 0.0001), cN factor (N0 vs. N1 o r2, *p* = 0.0094), and LNR (≤0.22 vs. >0.22, *p* = 0.0056) were significant prognostic factors (Table 2). A multivariate analysis showed that the cN stage (*p* = 0.0143) and LNR (*p* = 0.0071) were significant independent prognostic factors for patients with pathological N2 NSCLC (Table 3). The 5-year survival rate after surgery according to the cN stage (N0 and N1–2) was 39.5 and 21.2%, respectively (Fig. 1a). The 5-year survival rate of patients with an LNR of ≤0.22 was 40.2%; however, that of patients with pathological N2 having an LNR of ≥0.22 showed a statistically significant poorer survival rate (Fig. 1b). Figure 2 shows the comparison of survival curves among the subgroup on the bias of cN stage and LNR. Patients who had cN0 and an LNR of ≤0.22 showed a significantly higher survival rate than patients with cN1-2 and LNR of ≥0.22 and the 5-year survival rates were 47.1 and 10.3%, respectively (*p* < 0.0001).

**Table 2:** Survival of patients with N2 non-small cell lung cancer by a univariate analysis (log-rank test)

Variables		5-year survival (%)	*p* value
Gender			
	Male	29.6	0.083
	Female	37.7	
Side			
	Right	32.5	0.6669
	Left	27.3	
T factor			
	T1-2	34.8	<0.0001
	T3	8.9	
Histology			
	Adenocarcinoma	34.3	0.0856
	Others	23.0	
cN			
	N0	39.5	0.0094
	N1-2	21.2	
Skip metastasis			
	Skip	34.8	0.0848
	Non-skip	24.5	
Number of metastatic stations			
	Single	35.8	0.2739
	Multiple	27.7	
Lymph node ratio			
	≤0.22	40.2	0.0056
	>0.22	21.9

**Table 3:** Prognostic factors for overall survival retained in a multivariate analysis

Variables	Hazard ration	95% CI	*p* value
Pathological T factor	1.301	1.020–1.643	0.0531
Histology	0.932	0.546–1.466	0.571
Clinical N stage	1.386	1.047–1.512	0.0143
Skip metastasis	0.722	0.487–0.988	0.123
Lymph node ratio	1.725	1.174–2.152	0.0071

## Discussion

This retrospective study clarified the prognostic importance of the LNR in patients with pathological N2 NSCLC, who underwent complete dissection of the mediastinal lymph nodes. Our results indicated that LNR was an important prognostic factor for poor outcome after surgery in patients with N2 disease.

The 5-year survival rate for patients with p-stage III was 33.4% in the current series, which was consistent with the Japanese Lung Cancer Registry Study results. Several factors such as cN factor, N2 level, tumor size, tumor location, and skip N2 are important postoperative prognostic factors in patients with N2 disease [2, 6–10]. A single N2 disease showed favorable prognosis compared to those with multiple N2 disease [2, 11], and skip metastatic disease is a favorable N2 subset, possibly because it is usually associated with single-level N2 metastatic involvement [8, 12]. The present study demonstrated that skip metastasis or single N2 disease showed favorable prognoses, however, these differences were not statistically significant. On the other hand, Benoit et al. [13] reported that skip metastases occur frequently in NSCLC and complete dissection of the hilar and mediastinal lymph node should remain the standard surgical procedure for this disease. However, skip metastasis is not an independent prognostic factor for survival. Bria et al. [14] used the number of high risk factors (HRFs) as the standard to divide patients into risk classes. HRFs included the LNR, sex, stage, N status, grade, histology, age, and the number of involved nodes.

In this study, cN stage was an independent prognostic factor for patients with pathological N2 disease. Many previous studies have reported significant associations with survival for cN factors in patients with stage IIIA NSCLC [2, 15, 16]. In our study, the mediastinal lymph node sizes by CT scan and/or positive uptake by FDG-PET were diagnosed as metastatic lymph nodes. Prenzel et al. [17] described the difficulty of defining cut-off values to diagnose metastasis, and also showed a significant difference between the sizes of non-metastatic lymph nodes and infiltrated nodes. PET scanning is highly sensitive for the detection of mediastinal metastasis [18]. A controlled multicenter clinical study reported that FDG-PET improved diagnosis precision for mediastinal lymph nodes [19]. One of the limitations of the present study was that PET-scanning was initiated in 2004, and not all cases were examined using this method.

The number of involved lymph nodes that were identified depended on the number of lymph nodes removed and examined, which in itself depended on surgical and pathological procedures. In cases where few nodes were removed, the N stage could not be accurately classified. To improve the prognostication system, the LNR, which takes into account not only the number of positive nodes but also the number of nodes examined, removed the variability in nodal assessment.

Our results were also consistent with the findings of several recent studies evaluating the relationship between the LNR and survival for colon, breast, gastric, and bladder cancers, which further support the validity of our findings. The most recent TNM staging system for breast and gastric cancers suggested that the number of involved nodes has a significant prognostic value **[** 20–23].

Limitations of the present study included the retrospective nature of the analysis, and adjuvant chemotherapy for N2 disease was not routinely performed for all patients. Therefore, it was difficult to evaluate the effect of adjuvant chemotherapy on prognosis.

## Conclusions

In conclusion, data regarding the LNR or cN status could be used to provide a more accurate prognosis in patients with resected N2 NSCLC. The combination of the LNR and cN status provides a valuable prognostic tool. These findings have potential for predicting the best therapeutic modalities for patients with pathological N2 disease.

## Abbreviations

cN: Clinical N lymph node stages
CT: Computed tomography
FDG-PET: Fluoro-deoxyglucose-positron emission tomography
HRFs: High risk factors
LNR: Lymph node ratio
MRI: Magnetic resonance imaging
NSCLC: Non-small cell lung cancer
TNM: tumor node metastasis

## References

[1] Andre F, Grunenwald D, Pignon JP, Dujon A, Pujol JL (2000). Survival of patients with resected N2 non-small cell lung cancer: evidence for a subclassification and implications. J Clin Oncol.

[2] Inoue M, Sawabata N, Takeda S, Ohta M, Ohno Y (2004). Results of surgical intervention for p-stage IIIA (N2) non-small cell lung cancer: acceptable prognosis predicted by complete resection in patients with single N2 disease with primary tumor in the upper lobe. J Thorac Cardiovasc Surgery.

[3] Suzuki K, Nagai K, Yoshida J, Nishimura M, Takahashi K (1999). The prognosis of surgically resected N2 non-small cell lung cancer: the importance of clinical N status. J Thorac Cardiovasc Surg.

[4] Wisnivesky JP, Arciniega J, Mhango G, Mandeli J, Halm EA (2011). Lymph node ratio as a prognostic factor in elderly patients with pathological N1 non-small cell lung cancer. Thorax.

[5] Wang CL, Li Y, Yue DS, Zhang LM, Zhang ZF (2012). Value of the metastatic lymph node ratio for predicting the prognosis of non-small cell lung cancer patients. World J Surg.

[6] Watanabe Y, Hayashi Y, Shimizu J, Oda M, Iwa T (1991). Mediastinal nodal involvement and prognosis of non-small cell lung cancer. Chest.

[7] Ichinose Y, Kato H, Koike T, Tsuchiya R, Fujisawa T (2001). Completely resected stage IIIA non-small cell lung cancer: the significance of primary tumor location and N2 station. J Thorac Cardiovasc Surg.

[8] Riquet M, Assouad J, Bagan P, Foucault C, Le Pimpec BF (2005). Skip mediastinal lymph node metastasis and lung cancer: a particular N2 subgroup with a better prognosis. Ann Thorac Surg.

[9] Mountain CF, Dresler CM (1997). Regional lymph node classification for lung cancer staging. Chest.

[10] Naruke T, Suemasu K, Ishikawa S (1978). Lymph node mapping and curability at various levels of metastasis in resected lung cancer. J Thorac Cardiovasc Surg.

[11] Misthos P, Sepsas E, Kokotsakis J, Skottis I, Lioulias A (2008). The significance of one-station N2 disease in the prognosis of patients with non-small cell lung cancer. Ann Thorac Surg.

[12] Prenzel KL, Monig SP, Sinning JM, Baldus SE, Gutshow CA (2003). Role of skip metastasis to mediastinal lymph nodes in non-small cell lung cancer. J Surg Oncol.

[13] Benoit L, Anusca A, Ortega-Deballon P, Cheynel N, Bernard A (2006). Analysis of risk factors for skip lymphatic metastasis and their prognostic value in operated N2 non-small-cell lung carcinoma. Eur J Surg Oncol.

[14] Bria E, Milella M, Sperduti I, Alessandrini G, Visca P (2009). A novel clinical prognostic score incorporating the number of resected lymph nodes to predict recurrence and survival in non-small cell lung cancer. Lung Cancer.

[15] Sakao Y, Miyamoto H, Yamazaki A, Oh T, Fukai R (2006). Prognostic significance of metastasis to the highest mediastinal lymph node in non-small cell lung cancer. Ann Thorac Surg.

[16] Saito M, Kato H (2008). Prognostic factor in patients with pathological and N2 non-small cell lung cancer. Ann Thorac Cardiovasc Surg.

[17] Prenzel KL, Monig SP, Sinning JM, Baldus SE, Brochhagen HG (2003). Lymph node size and metastatic infiltration in non-small cell lung cancer. Chest.

[18] Ung YC, Mazlak DE, Vanderveen JA, Smith CA, Gulenchyn K (2007). Lung Cancer Disease Site Group of Cancer Care Ontarios Program in Evidence-Based Care. 18Fluorodeoxyglucose positron emission tomography in the diagnosis and staging of lung cancer: a systematic review. J Natl Cancer Inst.

[19] Kubota K, Murakami K, Inoue T, Itoh H, Saga T (2011). Additional value of FDG-PET to contrast enhanced computed tomography (CT) for the diagnosis of mediastinal lymph node metastasis in non-small cell lung cancer: a Japanese multicenter clinical study. Ann Nucl Med.

[20] Kodera Y, Yamamura Y, Shimizu Y, Torii A, Hirai T (1998). The number of metastatic lymph nodes: a promising prognostic determinant for gastric carcinoma in the latest edition of the TNM classification. J Am Coll Surg.

[21] Ichikura T, Tomimatsu S, Uefuji K, Kimura M, Uchida T (1999). Evaluation of the new American joint committee on cancer/international union against cancer classification of lymph node metastasis from gastric carcinoma in comparison with the Japanese classification. Cancer.

[22] Benson JR, Weaver DL, Mittra I, Hayashi M (2003). The TNM staging system and breast cancer. Lancet Oncol.

[23] Sinn HP, Helmchen B, Wittekind CH (2010). TNM classification of breast cancer: changes and comments on the 7^th^ edition. Pathologe.

